# Predicting complex quantitative traits with Bayesian neural networks: a case study with Jersey cows and wheat

**DOI:** 10.1186/1471-2156-12-87

**Published:** 2011-10-07

**Authors:** Daniel Gianola, Hayrettin Okut, Kent A Weigel, Guilherme JM Rosa

**Affiliations:** 1 Dept. of Animal Sciences, University of Wisconsin, Madison, 53706, USA; 2 Dept. of Dairy Science, University of Wisconsin, Madison, 53706, USA; 3 Dept. of Biostatistics and Medical Informatics, University of Wisconsin, Madison, 53706, USA; 4 Dept. of Animal Sciences, Biometry and Genetics Branch, University of Yuzuncu Yil, Van, 65080, Turkey

## Abstract

**Background:**

In the study of associations between genomic data and complex phenotypes there may be relationships that are not amenable to parametric statistical modeling. Such associations have been investigated mainly using single-marker and Bayesian linear regression models that differ in their distributions, but that assume additive inheritance while ignoring interactions and non-linearity. When interactions have been included in the model, their effects have entered linearly. There is a growing interest in non-parametric methods for predicting quantitative traits based on reproducing kernel Hilbert spaces regressions on markers and radial basis functions. Artificial neural networks (ANN) provide an alternative, because these act as universal approximators of complex functions and can capture non-linear relationships between predictors and responses, with the interplay among variables learned adaptively. ANNs are interesting candidates for analysis of traits affected by cryptic forms of gene action.

**Results:**

We investigated various Bayesian ANN architectures using for predicting phenotypes in two data sets consisting of milk production in Jersey cows and yield of inbred lines of wheat. For the Jerseys, predictor variables were derived from pedigree and molecular marker (35,798 single nucleotide polymorphisms, SNPS) information on 297 individually cows. The wheat data represented 599 lines, each genotyped with 1,279 markers. The ability of predicting fat, milk and protein yield was low when using pedigrees, but it was better when SNPs were employed, irrespective of the ANN trained. Predictive ability was even better in wheat because the trait was a mean, as opposed to an individual phenotype in cows. Non-linear neural networks outperformed a linear model in predictive ability in both data sets, but more clearly in wheat.

**Conclusion:**

Results suggest that neural networks may be useful for predicting complex traits using high-dimensional genomic information, a situation where the number of unknowns exceeds sample size. ANNs can capture nonlinearities, adaptively. This may be useful when prediction of phenotypes is crucial.

## Background

Challenges in the study of associations between genomic variables (e.g., molecular markers) and complex phenotypes include the possible existence of cryptic relationships that may not be amenable to parametric statistical modeling, as well as the high dimensionality of the data, illustrated by the growing number of single nucleotide polymorphisms, now close to 10 million in humans http://www.genome.gov/11511175. These associations have been investigated primarily using naïve single-marker regressions and, more recently, with Bayesian linear regression models of various types [1-3] but that assume additive inheritance almost invariably, while typically ignoring interactions and non-linearity. Taking into account these phenomena may enhance the ability of predicting outcomes, and this is relevant in genome-assisted management of livestock and plants and in individualized medicine.

There has been a growing interest in the use of non-parametric methods for prediction of quantitative traits based on reproducing kernel Hilbert spaces regressions on markers [2,4-7] and radial basis functions models [8] or related approaches [9]. Artificial neural networks (ANN) provide an interesting alternative because these learning machines can act as universal approximators of complex functions [10,11]. ANNs can capture non-linear relationships between predictors and responses and learn about functional forms in an adaptive manner, because a series of transformations called activation functions are driven by parameters. ANNs can be viewed as a computer based system composed of many processing elements (neurons) operating in parallel [12], and also as a schematic of Kolmogorov's theorem for representation of multivariate functions [13]. An ANN is determined by the network structure, represented by the number of layers and of neurons, by the strength of the connections (akin to non-parametric regression coefficients) between inputs, neurons and outputs, and by the type of processing performed at each neuron, represented by a linear or non-linear transformation: the activation function. Neural networks have the potential of accommodating complex relationships between input and response variables, as well as of difficult to model interactions among inputs. For these reasons, ANNs are interesting candidates for the analysis of complex traits affected by cryptic forms of gene X gene interaction, and many algorithms for training (fitting) such networks are now available [14].

In this study we investigated the performance of several ANN architectures using Bayesian regularization (a method for coping with the "small *n*, large *p*" problem that arises in statistical models including a massive number of explanatory variables) when predicting milk production traits in a sample of Jersey cows or mean grain yield in hundreds of inbred wheat lines. The architectures considered differed in terms of number of neurons and activation functions used, and the input (predictor) variables were derived from pedigree and molecular marker information on the corresponding samples. The paper begins with a brief account of Bayesian regularized neural networks, of their connection with linear random regression models often used in quantitative genetics, and of how Bayesian regularization is made. Subsequently, it is shown how a neural network treatment of genomic data can enhance predictive ability over and above that using pedigree information (in Jerseys) or linear Bayesian regression on markers (in both cows and wheat), which is representative of a standard approach in quantitative genomics.

## Methods

For clarity of presentation the methodology is presented first, as the main objective of the paper was to cast neural networks in a quantitative genetics predictive context. Subsequently, a description of the two sets of data used to illustrate how the Bayesian neural networks were run is provided. As stated, the first data set consisted of milk, protein and fat yield in dairy cows. The second set represented 599 lines of wheat, with mean grain yield as target trait.

### Excursus: Feed-Forward Neural Networks

**Figure 1:**
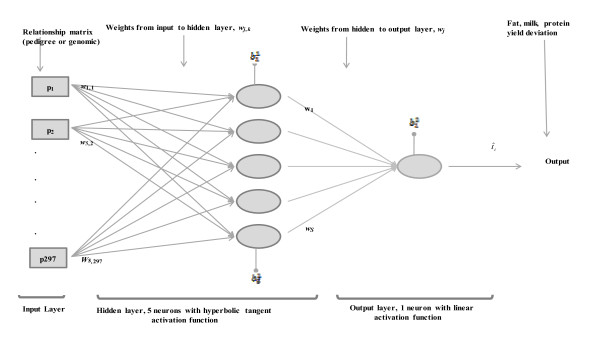
**Illustration of the neural networks used**. In the Jersey data there were 297 elements of pedigree or genomic relationship matrices used as inputs (the *p's*) for each target trait. In the Figure, each *p_k _(k = 1,2,...,297) *is connected to 5 hidden neurons via coefficients *w_j,k _*(*j *denotes neuron, *k *denotes input). Each hidden and output neuron has a bias parameter $b_{j}^{\left(l\right)}$, *j *denotes neuron, *l *denotes layer). The variable ${\hat{t}}_{i}$ represents the trait predicted value for datum *i*.

To illustrate, consider a network with three layers, as shown in Figure 1 for the Jersey cow data. In the left-most layer, there are input variables, 297 in Figure 1, or transformations thereof (called features) that enter into the network as predictors. In the middle ("hidden") layer there is a varying number of neurons; 5 are shown in Figure 1, but the number used is a model selection issue, with this addressed via an evaluation of predictive performance. In the right-most layer, there is a single ("output") node, at least for quantitative response variables. Each input (or feature) connects to each neuron with a strength represented by an unknown coefficient *w*. The collected input into a given neuron can be transformed (or not, in which case one speaks of an identity or linear activation function), and this activated net input is emitted to the output layer with a strength represented by another unknown coefficient. A similar process takes place for every neuron.

Algebraically, the process can be represented as follows. Let *t_i _*(the target phenotype) be a quantitative trait measured in individual *i *(*i = *1,2,...,n) and let *p_i _*= {*p_ij_*} be a vector of inputs or explanatory variables, e.g., marker genotypes or any other covariate measured in each of such individuals, with allowance made for inclusion of a 1, corresponding to the indicator variable for an intercept in a regression model. Suppose there are *S *neurons in the hidden layer of the architecture. The input into neuron *k *(*k *= 1,2,...,*S*) prior to activation, as described subsequently, is the linear function *w'_k _p_i_*, where *w'_k _*={*w_kj _*} is a vector of unknown connection strengths ("regressions") peculiar to neuron *k*, including an intercept (called "bias" in the machine learning literature) in *w'_k_*. This input is transformed ("activated") using some linear or non-linear function *f(.)*, which can be neuron-specific or common to all neurons; this yields *f_k_*(*w'_k _p_i_*) (*k *= 1,2,...,S). Subsequently, the so activated emission from neuron *k *is sent to the output layer, with the collection of emissions over all neurons being $b+cg\left[\overset{s}{\underset{k=1}{\sum }}w_{k}f_{k}\left({{\text{w}}^{\prime }}_{k}{\text{p}}_{i}\right)\right]$, where *b *is an overall bias parameter, *c *is a regression on an activated emission, *g(.) *is another activation function, possibly non-linear, and *w_1_,w_2_,...,w_S _*are regressions on each of the activated emissions *f_k_*(w'_k _p_i_). The link between the response variable (phenotype) and the inputs is provided by the model

(1) $$t_{\text{i}}=b+cg\left[\overset{s}{\underset{k=1}{\sum }}w_{\text{k}}f_{k}\left({\text{w}}_{\text{k}}^{\prime }{\text{p}}_{\text{i}}\right)\right]+e_{i};\;i=1,2,\ldots ,n$$

where *e_i _*~ (0, *σ*^2^) and *σ*^2 ^is a variance parameter. If *g*(.) is a linear or identity activation function, the model is a linear regression on the adaptive covariates *f_k_*(*w'_k _p_i_*); if, further, *f_k_*(.), is also linear, the regression model is entirely linear. The term "adaptive" means that the covariates are functions of unknown parameters, the {*w_kj_*} connection strengths, so the networks can "learn" the relationship between explanatory variables and phenotypes, as opposed to posing it arbitrarily, as it is the case in standard regression models. In this manner, this type of neural network can also be viewed as a regression model, but with the extent of non-linearity dictated by the type of activation functions used. Since the number of parameters increases linearly with the number of neurons, and the number of predictors given by the length of *p *(e.g., the number of markers) can amply exceed sample size, it is necessary to treat the connection strengths as random effects in which case the Bayesian connection is immediate [15,16]. This approach is called "Bayesian regularization".

### Fisher's infinitesimal model viewed as a neural network

Let *t *represent an *n *× 1 vector of phenotypic values and **u **~ (**0**, **A***σ*^2^*u *) be a vector of infinitesimal additive genetic effects, where *σ*^2^*u *is the additive genetic variance, ***A = CC' ***= {*a_ij_*} is the numerator relationship matrix and ***C ***is its lower triangular Cholesky factor decomposition. Fisher's linear model on additive genetic effects (ignoring an overall mean and nuisance fixed effects, for simplicity) admits at least three representations:

I) **t **= **u **+ **e **= **Cz***σ_u _*+ **e **= Cu* + **e**,

where **z **is a vector of independent standard normal deviates, u* = **z***σ_u _*~ (**0**, **I***σ*^2^*_u_*) and **e **~ (**0**, **I***σ*^2^) is a residual vector with *σ*^2 ^interpretable as environmental variance.

II) **t **= **AA**^-**1**^**u **+ e = Au** + **e**,

where u** = **A**^-**1**^**u **~ (**0, A**^-1 ^*σ*^2^*_u_*), and

III) **t **= **A**^-**1 **^**Au + e = A**^-**1**^u*** + **e**,

where u*** = **Au ~ **(**0**, **A**^3^*σ*^2^*u*).

In each of these formulations Fisher's model can be viewed as a neural network with a single neuron in the middle layer, where *g(.) *is an identity or linear activation function. The respective representations for the three models given above are

$$\begin{array}{c} t_{i}=b+g\left(\sum_{j=1}^{n}c_{ij}u_{j}^{*}\right)+e_{i},\\ t_{i}=b+g\left(\sum_{j=1}^{n}a_{ij}u_{j}^{**}\right)+e_{i}, \end{array}$$

and

$$t_{i}=b+g\left(\sum_{j=1}^{n}a^{ij}u_{j}^{***}\right)+e_{i}.$$

Here, a bias parameter *b *is included for the sake of generality. Hence, the additive model can be viewed as a single-neuron network regression on either elements of the Cholesky decomposition of the numerator relationship matrix, on the relationships themselves or on the elements of the inverse of **A**, with the strengths of the connections represented by the corresponding entries of u*, u** and u***, respectively.

Is it possible to exploit knowledge of relationships in a fuller manner? Since a neural network is a universal approximator, the predictive performance of the classical infinitesimal linear model can be enhanced, at least potentially, by taking a model on, say, *S *neurons, while effecting non-linear transformations simultaneously. The rationale is that Fisher's model holds under some assumptions which may be violated, such as linkage equilibrium, e.g., entries of the numerator relationship matrix are expected values in the absence of selection and under linkage equilibrium. For instance, using the second representation above one could write

(2) $$t_{\text{i}}=b+cg\left[\overset{s}{\underset{k=1}{\sum }}w_{k}g_{k}\left(b_{k}+\sum_{j=1}^{n}a_{ij}u_{j}^{**\left[k\right]}\right)\right]+e_{i};\;i=1,2,\ldots ,n.$$

Here, the inputs are entries *a_ij _*of the relationship matrix, connecting individual *i *to all other individuals in the genealogy; the $u_{j}^{**\left[k\right]}$ coefficient is the connection strength for input *j *in neuron *k; b_k _*is the bias parameter associated with neuron *k; g_k _*is an activation function peculiar to neuron *k; w_k _*is the connection strength between the activated emission from neuron *k *and the output layer, *b *is the outer layer bias parameter and *g(.) *is the outer activation function, which may be linear or non-linear, although it is typically taken as linear for quantitative responses. The nonlinear transformations modify the connection strengths between additive relationships and phenotypes in an adaptive manner, underlining the potential for an improvement in predictive ability.

Given the availability of dense markers in humans and animals, an alternative or complementary source of input that can be used in equation (2) consists of the elements of a marker-based relationship matrix, as in [17]; in this case the *a_ij _*coefficients are replaced by *g_ij_*, i.e., elements of some genome or marker-derived relationship matrix **G**. As noted by [2], when **G **is proportional to **XX'**, where **X **is the incidence matrix of a linear regression model on markers, this is equivalent to Bayesian ridge regression. Of course, nothing precludes using both pedigree-derived and marker-derived inputs in the construction of a neural network.

### Bayesian regularization

The objective in ANNs is to arrive at some configuration that fits the training data well but that it also has a reasonable ability of predicting yet to be seen observations. This can be achieved by placing constraints on the size of the network connection strengths, e.g., via shrinkage, and the process is known as regularization. A natural way of attaining this compromise between goodness of fit and predictive ability is by means of Bayesian methods [2,11,15,18]. In this section, an approach used often for Bayesian regularization in neural networks [18,19] is presented along the lines of the hierarchical models employed by quantitative geneticists [15].

Conditionally on *m *network parameters, the *n *phenotypes or outputs (represented as *D *for data) are assumed to be mutually independent, with density function (inputs p are omitted in the notation)

(3) $$p\left(D|b,w,\sigma^{2},M\right)=\overset{n}{\underset{i=1}{\prod }}N\left(t_{i}|b,w,\sigma^{2},M\right)$$

where *N(.) *denotes a normal density; *b *is the outer bias parameter; **w **denotes all connection strength coefficients (including all neuron-specific biases); *σ^2 ^*is the residual variance and *M *represents a given neural network architecture (i.e., a choice of number of neurons and activation functions). The mean of this distribution is the conditional (given all regression coefficients) expectation function $b+cg\left[\overset{s}{\underset{k=1}{\sum }}w_{k}g_{k}\left(b_{k}+\sum_{j=1}^{n}a_{jj}u_{j}^{**\left[k\right]}\right)\right]$, *i *= 1,2,...,*n*. The bias parameter *b *can be eliminated simply by taking deviations from the mean, or assigned a flat prior; for simplicity the first of the two options was employed in this study. The Bayesian approach used in regularized neural networks software (e.g., MATLAB) assigns the same normal prior distribution to each of the connection strengths, assumed independent a priori, such that *p*(**w**| *σ*^2^*_w_*) = *N*(0, **I***σ*^2^*_w_*), where *σ*^2^*_w _*is the variance of connection strengths. More general specifications can be posed, but currently available software (public or commercially) lacks flexibility for doing so. Assuming that the two variance parameters are known, the posterior density of the connection strengths is

(4) $$P\left(w|D,\sigma^{2},\sigma_{w}^{2},M\right)=\frac{P\left(D|w,\sigma^{2},M\right)P\left(w|\sigma_{w}^{2},M\right)}{P\left(D|\sigma^{2},\sigma_{w}^{2},M\right)},$$

where the denominator is the marginal density of the data, that is

$$P\left(D|\sigma^{2},\sigma_{w}^{2},M\right)=\int P\left(D|w,\sigma^{2},M\right)P\left(w|\sigma_{w}^{2},M\right)dw.$$

For a neural network with a least one non-linear activation function, the integral is expressible as

(5) $$\begin{array}{l} p(D|\sigma^{2},\sigma_{w}^{2},M)={\left(\frac{1}{2\pi \sigma^{2}}\right)}^{\frac{n}{2}}{\left(\frac{1}{2\pi \sigma_{w}{}^{2}}\right)}^{\frac{m}{2}}\times \\ \int \text{exp}\left[-\frac{1}{2\sigma^{2}}\sum_{i=1}^{n}(t_{i}-b-cg[\sum_{k=1}^{s}w_{k}g_{k}(b_{k}+\sum_{j=1}^{n}a_{ij}u_{j}^{**[k]})\right])-\frac{1}{2\sigma_{w}^{2}}w'w]dw \end{array}$$

which does not have closed form, because of non-linearity. Recall that *b *can be set to 0 provided the observations are suitably centered.

Although a Bayesian neural network can be fitted using Markov chain Monte Carlo sampling, the computations are taxing because of the enormous non-linearities present coupled with the high-dimensionality of **w**, such as it is the case with genomic data. An alternative approach is based on computing conditional posterior modes of connection strengths, given some likelihood-based estimates of the variance parameters, i.e., as in best linear unbiased prediction (when viewed as a posterior mode) coupled with restricted maximum likelihood (where estimates of variances are the maximizers of a marginal likelihood). The conditional (given *σ*^2 ^and $\sigma_{w}^{2}$) log-posterior density of **w **is from equation (4)

$$L\left(w|D,\sigma^{2},\sigma_{w}^{2},M\right)=K+\log P\left(D|w,\sigma^{2},M\right)+\log P\left(w|\sigma_{w}^{2},M\right).$$

Let $\beta =\frac{1}{2\sigma^{2}}$ and $\alpha =\frac{1}{2\sigma_{w}^{2}}$ (a standard notation in neural networks literature), and

(6) $$\begin{array}{lll} F\left(\alpha ,\beta \right) & =\beta \overset{n}{\underset{i=1}{\sum }}{\left(t_{i}-b-cg\left[\overset{s}{\underset{k=1}{\sum }}w_{k}g_{k}\left(b_{k}+\sum_{j=1}^{n}a_{ij}u_{j}^{**\left[k\right]}\right)\right]\right)}^{2}+\alpha w^{\prime }w,\; & \text{(1)}\\ & =\beta E_{D}+\alpha E_{w}\; & \text{(2)}\\ & \; & \text{(3)} \end{array}$$

where

$$E_{D}={\sum_{i=1}^{n}\left(t_{i}-b-cg\left[\sum_{k=1}^{s}w_{k}g_{k}(b_{k}+\sum_{j=1}^{n}a_{ij}u_{j}^{**[k]}\right]\right)}^{2},$$

and *E_w _*= **w'w**. It follows that maximizing $L\left(w\;|D,\sigma^{2},\sigma_{w}^{2},M\right)$ is equivalent to minimizing *F*(*α*, *β*). This function is often referred to as a "penalized" sum of squares, and it embeds a compromise between goodness of fit, given by the sum of squares of the residuals *E_D_*, and the degree of model complexity, given by the sum of squares of the network weights *E_w_*. The value w = w^MAP ^that maximizes $L\left(\text{w}\;|D,\sigma^{2},\sigma_{w}^{2},M\right)$ is the mode of the conditional (given the variances) posterior density of the connection strengths; **MAP **stands for "*maximum a posteriori*".

If the additive infinitesimal model is represented as a neural network, the coefficient of heritability is given by $h^{2}=\frac{1}{2\alpha }∕\left(\frac{1}{2\alpha }+\frac{1}{2\beta }\right)=\frac{\beta }{\alpha +\beta }$. As it can be seen in equation (6), if α<<β, the fitting or training algorithm places more weight on goodness of fit. If α>>β, the algorithm emphasizes reduction in the values of **w **(shrinkage), which produces a less wiggly output function [20].

Given α and β, the w = w^MAP ^estimates can be found via any non-linear maximization algorithm as in, e.g., the threshold and survival analysis models of quantitative genetics [21].

### Tuning parameters α and β

A standard procedure used in neural networks (and in the software employed here) infers α and β by maximizing the marginal likelihood of the data in equation (5); this corresponds to what is often known as empirical Bayes. Because (5) does not have a closed form (except in linear neural networks), the marginal likelihood is approximated using a Laplacian integration done in the vicinity of the current value w = w^MAP^, which depends in turn on the values of the tuning parameters at which the expansion is made. This type of approach for non-linear mixed models has been used in animal breeding for almost two decades [22].

The Laplacian approximation to the marginal density in equation (5) leads to the representation

(7) $$\begin{array}{c} \log\left[p\left(D|\alpha ,\beta ,M\right)\right]\approx K+\frac{n}{2}\log\left(\beta \right)+\frac{m}{2}\log\left(\alpha \right)\\ \;\;\;\;\;\;\;\;\;\;\;\;\;\;\;-{\left|F\left(\alpha ,\beta \right)\right|}_{w=w_{\left(\alpha ,\beta \right)}^{MAP}}-\frac{1}{2}\log{∥H∥}_{w=w_{\left(\alpha ,\beta \right)}^{MAP}} \end{array}$$

where *K *is a constant and $H=\frac{\partial^{2}}{\partial w\partial w^{\prime }}F\left(\alpha ,\beta \right)$ is the Hessian matrix. A grid search can be used to locate the α, β maximizers of the marginal likelihood in the training set. An alternative approach described by [18,23] involves the iteration (updating is from right to left, with **w**^MAP ^evaluated at the "old" values of the tuning parameters)

$$\alpha_{new}=\frac{m}{2\left({w^{MAP}}^{\prime }w^{MAP}+trH_{MAP}^{-1}\right)}$$

and

$$\beta_{new}=\frac{n-m+2\alpha_{MAP}trH_{MAP}^{-1}}{2\overset{n}{\underset{i=1}{\sum }}{\left(t_{i}-b-\overset{s}{\underset{k=1}{\sum }}w_{k}g_{k}\left(b_{k}+\sum_{j=1}^{n}a_{ij}u_{j}^{**\left[k\right]}\right)\right)}_{w=w_{\left(\alpha ,\beta \right)}^{MAP}}^{2}}$$

These expressions, as well as (7), are similar to those that arise in maximum likelihood estimation of variance components [24-26], which vary depending on the algorithm used. Since β is a positive parameter, it must be that $n>m-2\alpha_{MAP}trH_{MAP}^{-1}$. The quantity $\gamma =m-2\alpha_{MAP}trH_{MAP}^{-1}$ is called "effective number of parameters" in the network [20] and its value ranges from 0 (or 1, if an overall intercept *b *is fitted) to *m*, the total number of connection coefficients and bias parameters in the network. If γ is close to *n *over-fitting results, leading to poor generalization. It follows that the computations are similar to those used in the linear and non-linear models employed by quantitative geneticists, the salient aspect being that a neural network can be strongly non-linear.

More details on computing procedures for neural networks are in [12,14,18,23,27,28]. Typically, an algorithm proceeds as follows: 1) initialize *α*, *β *and all elements in w. 2) Take one step of the Levenberg-Marquardt algorithm to minimize the objective function *F(α, β) *and find the current value of **w**. 3) Compute *γ*, the effective number of parameters, using the Gauss-Newton approximation to the Hessian matrix in the Levenberg-Marquardt training algorithm. 4) Compute updates *α_new _*and *β_new_*; and 5) iterate steps 2-4 until convergence.

### Neural Network Architectures Evaluated and Implementation

A prototype of the networks considered is in Figure 1; as already noted, the architecture shown has five neurons in the hidden (middle) layer. The ANN examined had from 1 through 6 neurons in the hidden layer. In architectures with a single neuron, two variants were considered. In one, the activation functions were linear (identity) throughout. In this case, e.g., when additive relationships *a_ij _*are used as inputs, the network becomes a random regression on such relationships. If regularization were based on $w\sim N\left({0,A}^{-1}\sigma_{w}^{2}\right)$ as prior distribution, this would yield the standard additive ("animal") model of quantitative genetics; this was not the case here because the MATLAB software used (see below) bases regularization on $w\sim N\left(0,I\sigma_{w}^{2}\right)$. The second single-neuron architecture was based on equation (1) with a single outer bias parameter but with a non-linear activation *g *of the emission made by the sole neuron in the architecture. The algebraic representation of this network is

$$t_{\text{i}}=b+cg\left(b+\sum_{j=1}^{n}p_{ij}u_{j}^{**}\right)+e_{i},\;i=1,2,\ldots ,n,$$

where *c *is the regression of *t_i _*on the activated emission $g\left(b+\sum_{j=1}^{n}p_{ij}u_{j}^{**}\right)$. The objective here was to explore non-linearities between the inputs (additive or genomic relationships in the Jersey data, or markers genotypes in the wheat data) and the targets (phenotypes); the standard additive genetic model assumes that these relationships are linear. The activation function chosen was the hyperbolic tangent transformation $g\left(x_{i}\right)=\frac{e^{x_{i}}-e^{-x_{i}}}{e^{x_{i}}+e^{-x_{i}}}$, where $x_{i}=b+\sum_{j=1}^{n}p_{ij}u_{j}^{**}$; here, *x *can take any value in the real line and *g(x_i_) *is the neuron emission for cow or wheat line *i*, which takes values between -1 and 1. Given the inputs, the predicted phenotype or network output is

$${\hat{t}}_{i}=\hat{b}+ĉ\left(\frac{e^{\hat{b}+\Sigma_{j=1}^{n}p_{ij}u_{j}^{**}}-e^{-\hat{b}-\Sigma_{j=1}^{n}p_{ij}u_{j}^{**}}}{e^{\hat{b}+\Sigma_{j=1}^{n}p_{ij}u_{j}^{**}}+e^{-\hat{b}-\Sigma_{j=1}^{n}p_{ij}u_{j}^{**}}}\right)i=1,2,\ldots ,n.$$

In models with 2-6 neurons the emissions were always assigned a hyperbolic tangent activation (the choice of function can be based on, e.g., cross-validation); these activations were summed and collected linearly as shown in Figure 1. For example, with 2 neurons the predictions are obtained as

$$\begin{array}{c} {\hat{t}}_{i}=\hat{b}+{ĉ}_{1}\left(\frac{e^{{\hat{b}}_{1}+\Sigma_{j=1}^{n}p_{ij}u_{j}^{**\left[1\right]}}-e^{-{\hat{b}}_{1}-\Sigma_{j=1}^{n}p_{ij}u_{j}^{**\left[1\right]}}}{e^{{\hat{b}}_{1}+\Sigma_{j=1}^{n}p_{ij}u_{j}^{**\left[1\right]}}+e^{-{\hat{b}}_{1}-\overset{n}{\underset{j=1}{\sum }}p_{ij}u_{j}^{**\left[1\right]}}}\right)+\\ \;\;\;{ĉ}_{2}\left(\frac{e^{{\hat{b}}_{2}+\Sigma_{j=1}^{n}p_{ij}u_{j}^{**\left[2\right]}}-e^{{\hat{b}}_{2}-\Sigma_{j=1}^{n}p_{ij}u_{j}^{**\left[2\right]}}}{e^{{\hat{b}}_{2}+\Sigma_{j=1}^{n}p_{ij}u_{j}^{**\left[2\right]}}+e^{{\hat{b}}_{2}+\overset{n}{\underset{j=1}{\sum }}p_{ij}u_{j}^{**\left[2\right]}}}\right)\;i=1,2,\ldots ,n \end{array}$$

where the  coefficients are the estimated linear regressions of the traits on the activated emissions fired by each of the two neurons.

MATLAB's neural networks toolbox [29] was used for fitting the architectures studied, using Bayesian regularization in all cases. As mentioned earlier, two combinations of activation functions were tried: 1) the hyperbolic tangent sigmoid function for activating emissions from each neuron in the hidden layer, plus a linear activation function from the hidden to the output layer, and 2) a linear activation throughout, this corresponding to a linear model with random regression coefficients. To avoid spurious effects caused by starting values in each iterative sequence, the networks were trained 20 times in the Jersey data and 50 times in the wheat data set, for each of the architectures. In Jerseys, each run randomly allocated 60% of the animals to a training set, 20% to a validation set and 20% to a testing set; results reported are the average of the 20 runs for each of the configurations. In wheat, the records were randomly partitioned into a training (480 lines) and a testing (119 lines) set. Each of the 50 random repeats matched exactly those of [28], to provide a comparison with the predictive ability of the Bayesian Lasso and of support vector regression models used with the wheat data set by [30].

The neural networks were fitted to data in the training set, with the α and β parameters, connection strengths and biases modified iteratively. In the Jersey data, as parameters changed in the course of training, the predictive ability of the network was gauged in parallel in the validation set, which was expected to be similar in structure to the testing set, because they were randomly constructed. The same was done with the wheat data, except that there was no "intermediate" validation set. Once the mean squared error of prediction reached an optimal level, training stopped, and this led to the best estimates of the network coefficients. This estimated network was then used for predicting the testing set; predictive correlations (Pearson) and mean-squared errors were evaluated.

Before processing, MATLAB rescales all input and output variables such that they reside in the [-1, +1] range, to enhance numerical stability; this is done automatically using the "mapminmax" function. To illustrate, consider the vector **x**' = [3,6,4] so that *x*_min _= 3 and *x*_max _= 6. If values are to range between *A*_min _= -1 and *A*_max _= +1, one sets temporarily **x_temp_**' = [-1,1,4], so only *x_3 _= *4 needs to be rescaled. This is done via the formula

$$x_{3,new}=A_{\min}+\frac{x_{3}-x_{\min}}{x_{\max}-x_{\min}}\left(A_{\max}-A_{\min}\right)=-1+\frac{4-3}{6-3}2=-\frac{1}{3}.$$

MATLAB uses the Levenberg-Marquardt algorithm (based on linearization) for computing the posterior modes in Bayesian regularization, and back-propagation is employed to minimize the penalized residual sum of squares. The maximum number of iterations (called epochs) in back-propagation was set to 1000, and iteration stopped earlier if the gradient of the objective function was below a suitable level or when there were obvious problems with the algorithm [28,29,31]. Each of these settings corresponds to the default values provided by MATLAB.

#### Jersey cows data

Because of the high-dimensionality of the genotypic data, the neural networks used either additive or genome-derived relationships among cows as inputs (instead of SNP genotype codes), to make computations feasible in MATLAB. The rationale for this is based on the representation of the infinitesimal model as a regression on a pedigree, or as a regression on a matrix that is proportional to genomic relationships, as argued by [2] in the context of reproducing kernel Hilbert spaces regression. The neural networks had the form

(8) $$t_{\text{i}}=b+cg[\overset{s}{\underset{k=1}{\sum }}w_{k}g_{k}\left(b_{k}+\sum_{j=1}^{n}p_{ij}u_{j}^{**\left[k\right]}\right)+e_{i},\;i=1,2,\ldots ,n$$

where *p_ij _= a_ij _*(additive relationship between cows *i *and *j*) or *g_ij _*(genome-derived relationships). Thus, for each cow the input vector ***p**_i _*had order 297 × 1.

The expected additive genetic relationship matrix, **A **= {*a_ij_*}, was developed from the pedigree information; this is a standard metric for degree of kinship used in quantitative genetics. A realized genomic relationship matrix, **G **= {*g_ij_*}, was constructed from the marker data following [18], and calculated as follows: 1) estimate marker allelic frequencies and let *μ_i _*be the estimated frequency of allele "A" at locus *i*. 2) Construct a 297 × 35,798 matrix of marker genotype codes **M**, with typical element *m_ij _*corresponding to the genotype of individual *i *for marker *j*. 3) Calculate the expected frequency of *m_ij _*under Hardy-Weiberg equilibrium from the estimates of the allelic frequencies, and form the 297 × 35,798 matrix of expectations **E**. 4) Form the estimated genomic relationship matrix (assuming linkage equilibrium among markers) as

$$G=\frac{\left(M-E\right)\left(M-E\right)\text{’}}{2\overset{35,798}{\underset{l=1}{\sum }}\mu_{i}\left(1-\mu_{i}\right)}=\left\{g_{ij}\right\}.$$

The matrix **Z = M-E **contains "centered" codes, such that the mean of the values in any of its columns is null; **Z **can be used as in incidence matrix in marker assisted regression models [17,32,33]. Then $ZZ^{\prime }=G\times 2\overset{35,798}{\underset{i=1}{\sum }}\mu_{i}\left(1-\mu_{i}\right)$ is interpretable as a covariance matrix, analogous to $A\sigma_{u}^{2}$ in the infinitesimal model. The term $2\overset{35,798}{\underset{i=1}{\sum }}\mu_{i}\left(1-\mu_{i}\right)$ holds under linkage equilibrium only, and cannot be construed as additive genetic variance of marker effects in the classical sense of [33]; its relationship to additive genetic variance in a finite locus or infinitesimal model is tenuous [16,34].

#### Wheat lines data

There were 599 wheat lines, each genotyped with 1279 DArT markers (Diversity Array Technology) generated by Triticarte Pty. Ltd. (Canberra, Australia; http://www.triticarte.com.au). The DArT markers may take on two values, denoted by their presence or absence. In this data set, the overall mean frequency of the allele coded as "1" was 0.5607, with a minimum of 0.0083 and a maximum of 0.9866. Markers with a minor allele frequency lower than 0.05 were removed. Missing genotypes at locus *j *of line *i *were imputed using samples from the marginal distribution of marker genotypes, that is, $x_{ij}\sim Bernoulli\left({\hat{p}}_{j}\right)$, where ${\hat{p}}_{j}$ is the estimated allele frequency computed from the non-missing genotypes [34]. The phenotype studied was average grain yield of each line. The data came from the International Maize and Wheat improvement Center, Mexico, and it can be downloaded from R package BLR http://cran.r-project.org/web/packages/BLR/index.html; more information can be found in [30,35,36]. The wheat data was partitioned randomly into a training set (480 lines) and a test set (119 lines), exactly as in [30].

## Results

### Degree of complexity

**Figure 2:**
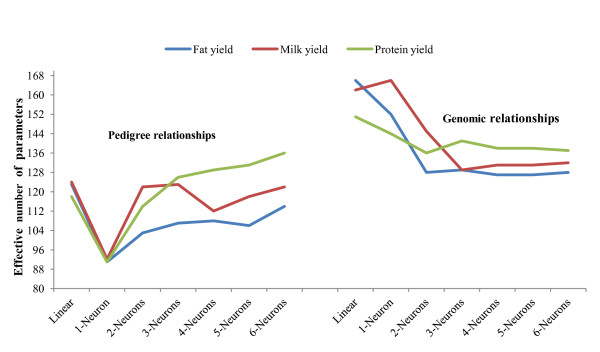
**Effective number of parameters obtained from different network architectures in the Jersey data**. Results shown are averages of 20 independent runs. "Linear" denotes a 1-neuron model with linear activation functions throughout.

The effective number of parameters (γ) associated with each of the networks examined in the Jersey data is presented in Table 1 and shown graphically in Figure 2, by trait and type of input considered, i.e., additive or genomic relationships. Clearly, use of genomic relationships resulted in a larger number of effective parameters than use of pedigree, for all traits and architectures. When using pedigree relationships, the average (over runs, but note the large standard errors) effective number of parameters ranged from 91 (fat yield, one-neuron model with non-linear activation), to 136 (protein yield, 6 neurons). This illustrates the impact of shrinkage, and of how regularized neural networks cope with the "curse of dimensionality. For example, a 6-neuron network has close to 1800 nominal parameters. Likewise, when using genomic relationships as inputs, the average effective number of parameters ranged from 127 to 166 (fat yield). Similar results were obtained in the wheat data (Table 2). The effective number of parameters ranged from 220 (nonlinear ANN with 4 neurons) to 299 (linear ANN).

**Table 1:** Effective number of parameters (± standard errors), by trait, in Jerseys.^1^

Network	Fat yield (pedigree)	Fat yield (genomic)	Milk yield (pedigree)	Milk yield (genomic)	Protein yield (pedigree)	Protein yield (genomic)
Linear	123 ± 5.6	166 ± 2.0	124 ± 7.6	162 ± 2.9	118 ± 8.5	151 ± 4.5
1 neuron	91 ± 4.9	142 ± 2.0	93 ± 5.8	166 ± 2.0	91 ± 10.3	144 ± 2.5
2 neurons	104 ± 5.8	128 ± 7.6	122 ± 6.5	145 ± 7.8	114 ± 8.0	136 ± 8.0
3 neurons	107 ± 5.8	132 ± 5.7	123 ± 5.1	129 ± 6.0	126 ± 6.9	141 ± 4.9
4 neurons	108 ± 5.8	129 ± 4.7	112 ± 4.7	131 ± 5.8	129 ± 5.4	138 ± 6.0
5 neurons	106 ± 4.9	127 ± 4.9	118 ± 4.8	132 ± 5.4	131 ± 4.9	138 ± 5.6
6 neurons	114 ± 3.3	128 ± 7.5	122 ± 5.1	132 ± 5.6	136 ± 4.6	137 ± 5.0

^1 ^Results are averages of 20 runs based on random partitions of the data

**Table 2:** Effective number of parameters, predictive correlations, and mean squared errors of prediction: wheat.^1^

ANN architectures	Linear	1 neuron	2 neurons	3 neurons	4 neurons
Criterion					

Effective number of parameters	299 ± 5.5	260 ± 6.1	253 ± 5.9	238 ± 5.5	220 ± 2.8
Correlations in testing set	0.48 ± 0.03	0.54 ± 0.03	056 ± 0.02	0.57 ± 0.02	0.59 ± 0.02
Mean squared error in testing set	0.99 ± 0.04	0.77 ± 0.03	0.74 ± 0.03	0.71 ± 0.02	0.72 ± 0.02

^1 ^The training-test partitions for this data were random and repeated 50 times; standard errors in parentheses

The effective number of parameters behaved differentially with respect to model architecture and this depended on the input variables used. When using pedigrees in the Jersey data, the hyperbolic tangent activation function in the 1-neuron model reduced γ drastically, relative to the linear model (1 neuron with linear activation throughout). Then, an increment in number of neurons from 2 to 6 increased model complexity relative to that of the 1 neuron model with non-linear activation, but not beyond that attained with the linear model, save for protein yield. For this trait, γ was 118 for the linear model, and ranged from 126 to 136 in models with 3 through 6 neurons. When genomic relationships were used as inputs, γ was largest for the linear model for fat and protein yield, and for the 1-neuron model with a non-linear activation function in the case of milk yield. In wheat, the effective number of parameters decreased as architectures became more complex. There was large variation among runs in effective number of parameters for both data sets, but there was not a clear pattern in the variability.

### Predictive performance

**Figure 3:**
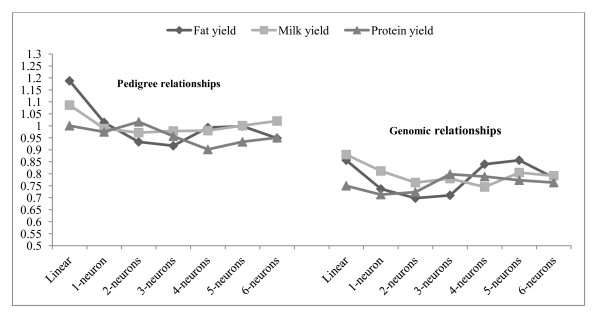
**Prediction mean squared errors in the Jersey testing set (vertical axis) by network**. Results are averages of 20 independent runs. "Linear" denotes a 1-neuron model with linear activation functions throughout.

**Figure 4:**
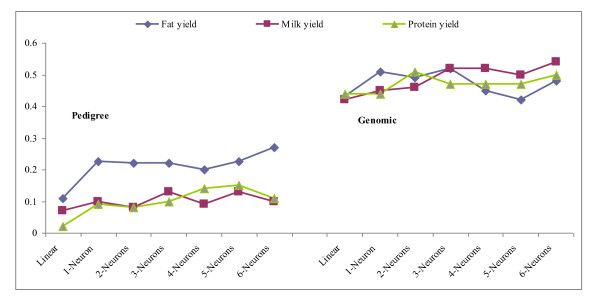
**Correlations between predictions and observations in the Jersey testing data set for the network considered**. Results shown are averages of 20 independent runs. "Linear" denotes a 1-neuron model with linear activation functions throughout.

Results pertaining to predictive ability evaluated in the testing sets are shown in Table 2 for wheat and Tables 3 and 4 for the Jersey data. Figures 3 and 4 depict mean of squared errors of prediction and correlations coefficients in the Jersey cows.

**Table 3:** Prediction mean squared errors (± standard errors) by trait: Jerseys.^1^

Network	Fat yield (pedigree)	Fat yield (genomic)	Milk yield (pedigree)	Milk yield (genomic)	Protein yield (pedigree)	Protein yield (genomic)
Linear	1.19 ± 0.07	0.86 ± 0.05	1.09 ± 0.05	0.88 ± 0.04	1.00 ± 0.04	0.75 ± 0.07
1 neuron	1.01 ± 0.04	0.74 ± 0.03	0.99 ± 0.04	0.81 ± 0.03	0.97 ± 0.04	0.71 ± 0.04
2 neurons	0.93 ± 0.05	0.70 ± 0.03	0.96 ± 0.05	0.76 ± 0.04	1.02 ± 0.04	0.72 ± 0.04
3 neurons	0.92 ± 0.04	0.71 ± 0.03	0.98 ± 0.02	0.78 ± 0.04	0.96 ± 0.06	0.80 ± 0.04
4 neurons	0.99 ± 0.04	0.84 ± 0.04	0.98 ± 0.04	0.72 ± 0.04	0.90 ± 0.06	0.70 ± 0.03
5 neurons	0.99 ± 0.04	0.86 ± 0.04	1.00 ± 0.05	0.80 ± 0.04	0.93 ± 0.04	0.77 ± 0.04
6 neurons	0.95 ± 0.03	0.77 ± 0.04	1.02 ± 0.05	0.79 ± 0.03	0.95 ± 0.03	0.76 ± 0.05

^1^Results are the average of 20 runs based on random partitions on the data

**Table 4:** Correlation coefficients (± standard errors) in the Jersey testing data set, by trait.^1^

	Pedigree relationships			Genomic relationships		
**Network**	**Fat yield**	**Milk yield**	**Protein yield**	**Fat yield**	**Milk yield**	**Protein yield**

Linear	0.11 ± 0.04	0.07 ± 0.03	0.02 ± 0.02	0.43 ± 0.02	0.42 ± 0.03	0.44 ± 0.02
1 neuron	0.23 ± 0.03	0.10 ± 0.03	0.09 ± 0.02	0.51 ± 0.02	0.45 ± 0.02	0.44 ± 0.02
2 neurons	0.22 ± 0.03	0.08 ± 0.01	0.08 ± 0.03	0.49 ± 0.02	0.46 ± 0.03	0.51 ± 0.02
3 neurons	0.22 ± 0.02	0.13 ± 0.02	0.10 ± 0.03	0.53 ± 0.02	0.52 ± 0.02	0.47 ± 0.02
4 neurons	0.20 ± 0.02	0.09 ± 0.02	0.14 ± 0.02	0.45 ± 0.03	0.52 ± 0.02	0.47 ± 0.03
5 neurons	0.23 ± 0.02	0.13 ± 0.02	0.15 ± 0.02	0.42 ± 0.03	0.50 ± 0.02	0.47 ± 0.02
6 neurons	0.27 ± 0.02	0.10 ± 0.03	0.11 ± 0.02	0.48 ± 0.04	0.54 ± 0.02	0.50 ± 0.03

^1^Results are the average of 20 runs based on random partitions on the data

The predictive correlations in wheat (Table 2) ranged from 0.48 with the linear ANN (equivalent to Bayesian ridge regression) to 0.59 for the nonlinear ANN with 4 neurons. Clear and significant differences between linear and nonlinear architectures were observed. The improvements over the linear ANN were 11.2, 14.3, 15.8 and 18.6% in predictive correlation for 1, 2, 3 and 4 neurons in the hidden layer, respectively. Mean squared errors were also 23-29% smaller than in the linear ANN.

In the Jerseys, the large variability in mean squared error among runs (Table 3) precludes making strong statements about differences among architectures. However, predictive correlations (Table 4) were clearly larger for the non-linear ANN. For fat yield, the results with pedigrees employed as input suggest that a non-linear, adaptive use of additive relationships (as done in all networks with the hyperbolic tangent activation function) can improve predictive performance beyond that of the infinitesimal model. Further, use of genomic relationships led to more reliable prediction of phenotypes than use of pedigree information as measured by the predictive correlations in Table 4. The relative increase in strength of association, as measured by the correlation, is much larger than the ones that have been reported, e.g., in dairy cattle [32,37], when prediction of breeding values of bulls was made from genomic information, as opposed to from pedigrees. Our result is encouraging and suggests that genomic data may also play an important role in prediction of individual outcomes (as opposed to breeding value), an issue of relevance in medicine [4].

### Shrinkage

**Figure 5:**
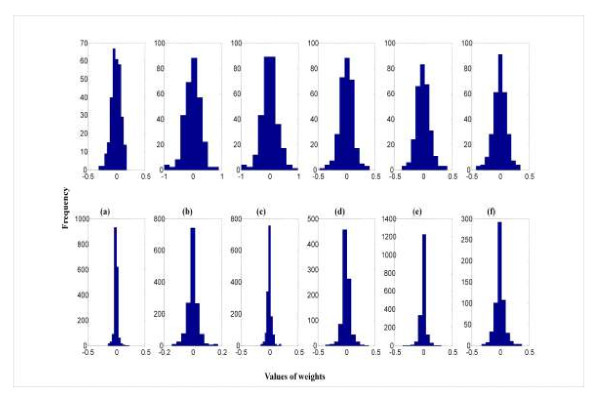
**Distribution of connection strengths(*w_kj_*) in the linear and selected networks fitted to the Jersey data**. The linear model has single neuron architecture with linear activation functions. a) Fat yield using pedigree relationships: linear model (above) and 6 neurons (below). b) Milk yield using pedigree relationships: linear model (above) and 6 neurons (below). c) Protein yield using pedigree relationships: linear model (above) and 5 neurons (below). d) Fat yield using genomic relationships: linear model (above) and 3 neurons (below), e) Milk yield using genomic relationships: linear model (above) and (below) and 6 neurons (below). f) Protein yield using genomic relationships: linear model (above) and 2 neurons (below).

The distribution of connection strengths in a network gives an indication of the extent of regularization attained. Typically, weight values decrease with model complexity, in the same manner that estimates of marker effects become smaller in Bayesian regression models when *p *increases and training sample size is kept constant. Further, the distribution of weights is often linked to predictive ability; small values tend to lead to better generalization. Figure 5 depicts the distributions of weights for the linear models and for the nonlinear regularized networks that produced the largest predictive correlations for pedigree and genomic relationships in the Jersey data. The weights for the linear model were larger and more variable than for the nonlinear networks, where distributions were patently leptokurtic, indicating strong shrinkage towards 0. For example, the average (over runs) sum of squares of weights for the linear model and for the non-linear network with 6 neurons when using genomic relationships as predictors of milk yield were 7.5 and 8.5, respectively; however, the 7.5 for the linear model was the sum of squares of 297 weights whereas the 8.5 for the nonlinear model with 6 neurons was the sum of squares of 1782 weights (6 × 297). The same picture was observed in the wheat data (results are not reported).

## Discussion

Models for prediction of fat, milk and protein yield in cows using pedigree and genomic relationship information as inputs, and wheat yield using molecular markers as predictor variables were studied. This was done using Bayesian regularized neural networks, and predictions were benchmarked against those from a linear neural network, which is a Bayesian ridge regression model. In the wheat data, the comparison was supplemented with results obtained by our group using RKHS or support vector methods. Different network architectures were explored by varying the number of neurons, and using a hyperbolic tangent sigmoid activation function in the hidden layer plus a linear activation function in the output layer. This combination has been shown to work well when extrapolating beyond the range of the training data [36]. The choice of number of neurons can be based on cross-validation, as in the present data, or on standard Bayesian metrics for model comparison [11,15].

The Levenberg-Marquardt algorithm, as implemented in MATLAB, was adopted to optimize weights and biases, as this procedure has been effective elsewhere [38]. Bayesian regularization was adopted to avoid over-fitting and to improve generalization, and cross-validation was used to assess predictive ability, as in [28,39].

Because Bayesian neural networks reduce the effective number of weights relative to what would be obtained without regularization, this helps to prevent over-fitting [40]. For the networks we examined, even though the total number of parameters, e.g., in Jerseys, ranged from 300 to 1795, the effective number of parameters varied from only 91 to 136 when pedigree relationships were used, and from 127 to 166 when genomic relationships were used as inputs, illustrating the extent of regularization. There were differences in predictive abilities of different architectures but the small sample used dictates a cautious interpretation. Nevertheless, the results seem to support networks with at least 2 neurons, which has been observed in several studies [20,28,41-43]. This suggests that linear models based on pedigree or on genomic relationships may not provide an adequate approximation to genetic signals resulting from complex genetic systems. Because highly parameterized models are penalized in the Bayesian approach, we were able to explore complex architectures. However, there was evidence of over-fitting in the Jersey training set, where correlations between observed and predicted data in the training set were always larger than 0.90, sometimes exceeding 0.95. This explains why correlations were much lower in the testing set, which is consistent with what was observed in other studies with neural networks [42]. Although more parameters in a model can lead to smaller error in the training data, it cannot be overemphasized that this is not representative of prediction error in an independent data set, as shown by [43] working with human stature.

Our results with ANN for wheat are at least as good as those obtained with the same data in two other studies. Crossa et al. [35] found cross-validation correlations with the following values when various methods were used: pedigree information (BLUP), 0.45; pedigree-based reproducing kernel Hilbert spaces regression (RKHS), 0.60; RKHS with both pedigree and markers, 0.61; Bayesian Lasso with markers, 0.46; Bayesian Lasso with markers and pedigree, 0.54, and Bayesian ridge regression on markers, 0.49. Long et al., [30] compared the Bayesian Lasso with four support-vector regression models consisting of the combination of two kernels and two loss functions. The predictive correlation for wheat yield (average of 50 random repeats of the cross-validation exercise) was 0.52 for the Bayesian Lasso, and ranged between 0.50 and 0.58 for the support vector implementations. Hence, it seems clear, at least for wheat yield in this data set, that the non-parametric methods can outperform a strong learner, the Bayesian Lasso, and that the neural networks are competitive with the highly regarded support vector methods [11].

A question of importance in animal and plant breeding is how an estimated "breeding value", i.e., an estimate of the total additive genetic effect of an individual, can be arrived at from an ANN output. There are at least two ways in which this can be done. One is by posing architectures with a neuron in which the inputs enter in a strictly linear manner, followed by a linear activation function on this neuron; the remaining neurons in the architecture, receiving the same inputs, would be treated non-linearly. A second one, is obtained by observing that the infinitesimal model can be written as y*i *= ***z***'*_i_***u **+ **e**, for some incidence row vector ***z***'*_i _*peculiar to individual *i*. Here, the breeding value of the *i^th ^*individual can be written as $u_{i}=z_{i}^{\prime }\frac{\partial }{\partial z_{i}}\left(z_{i}^{\prime }u\right)$. Likewise, consider a linear regression model for *p *markers, $y_{i}=\sum_{j=1}^{p}x_{ij}\beta_{j}+e_{i}=x_{i}^{\prime }\beta +e_{i}$, where *β_j _*is the substitution effect at marker locus *j*; *x_ij _*= 0,1,2 is the observed number of copies of a given allele at locus *j *on individual *I*, and $x_{i}^{\prime }=\left\{x_{ij}\right\}$ and ***β ***= {*β_j_*} are row and column vectors, respectively, each with *p *elements. Here, the "marked breeding value" of individual *i *would be $x_{i}^{\prime }\frac{\partial }{\partial x_{i}}\left(x_{i}^{\prime }\beta \right)=x_{i}^{\prime }\beta$. Consider next a neural network with a hyperbolic tangent activation function throughout, that is

$$t_{\text{i}}=b+cg\left[\overset{s}{\underset{k=1}{\sum }}w_{k}g_{k}\left(b_{k}+\sum_{j=1}^{n}p_{ij}u_{j}^{**\left[k\right]}\right)\right]+e_{i},.$$

Let ***p**_i _*= {*p_ij_*} be the vector of input covariates (e.g., genomic or additive relationships, marker genotype codes) observed on *i*. Adapting the preceding definitions to the ANN specification, one would have as breeding value (BV) of individual *i*

$$\begin{array}{c} BV_{i}={p^{\prime }}_{i}\frac{\partial }{\partial p_{i}}t_{i}=\\ C_{g^{\prime }}\left[\overset{s}{\underset{k=1}{\sum }}w_{k}g_{k}\left(b_{k}+\sum_{j=1}^{n}p_{ij}u_{j}^{**\left[k\right]}\right)\right]{p^{\prime }}_{\text{i}}\overset{s}{\underset{k=1}{\sum }}w_{k}{g^{\prime }}_{k}\left(b_{k}+\sum_{j=1}^{n}p_{ij}u_{j}^{**\left[k\right]}\right)u^{**\left[k\right]}, \end{array}$$

where:

$$\begin{array}{c} g^{\prime }\left[\overset{s}{\underset{k=1}{\sum }}w_{k}g_{k}\left(b_{k}+\sum_{j=1}^{n}p_{ij}u_{j}^{**\left[k\right]}\right)\right]=4P\left(1-P\right),\\ P=\frac{\exp\left[-2\overset{s}{\underset{k=1}{\sum }}w_{k}g_{k}\left(b_{k}+\sum_{j=1}^{n}p_{ij}u_{j}^{**\left[k\right]}\right)\right]}{1+\exp\left[-2\overset{s}{\underset{k=1}{\sum }}w_{k}g_{k}\left(b_{k}+\sum_{j=1}^{n}p_{ij}u_{j}^{**\left[k\right]}\right)\right]},\\ u^{**\left[k\right]}=\left\{u_{j}^{**\left[k\right]}\right\}, \end{array}$$

and

$${g_{k}}^{\prime }\left(b_{k}+\sum_{j=1}^{n}p_{ij}u_{j}^{**\left[k\right]}\right){u^{**}}^{\left[k\right]}=4P_{k}\left(1-P_{k}\right),$$

with

$$P_{k}=\frac{\exp\left[-2(b_{k}+\sum_{j=1}^{n}p_{ij}u_{j}^{**[k]}\right]}{1+\exp\left[-2(b_{k}+\sum_{j=1}^{n}p_{ij}u_{j}^{**[k]})\right]}.$$

Thus, the so defined breeding value of individual *i *depends on the values of the input covariates observed on this individual, on all connection strengths and bias parameters from inputs to neurons in the middle layer (the *u's *and the *b's*), and on all connection strengths from the middle layer to the output layer (the *w's*). In order to obtain an estimate of breeding value the unknown quantities would replaced by the corresponding maximum a posteriori (MAP) estimates or, say, by the estimate of their posterior expectation if a Markov chain Monte Carlo scheme is applied [44].

Another issue is that of assessing the importance of an input relative to that of others. For example, in a linear regression model on markers, one could use a point estimate of the substitution effect or its "studentized" value (i.e., the point estimate divided by the corresponding posterior standard deviation), or some measure that involves estimates of substitution effects and of allelic frequencies. A discussion of some measures of relative importance of inputs in an ANN is in [28,43], for example, the ratio between the absolute value of the estimate of a given connection strength, and the sum of the absolute values of all coefficients in the network.

## Conclusion

Non-linear neural networks tended to outperform benchmark linear models in predictive ability, and clearly so in the wheat data. Bayesian regularization allowed estimation of all connection strengths even when *n<<p*, and the effective number of parameters was much smaller than the corresponding nominal number. Although the study was based on small samples, and the differences found may be reflective of random variation, especially in the Jersey data, our results suggest that the neural networks may be useful for predicting complex traits using high-dimensional genomic information, a situation where the number of coefficients that need to be estimated exceeds sample size. Neural networks have the ability of capturing nonlinearities, and do so adaptively, which may be useful in the study of quantitative traits under complex gene action, and particularly when prediction of outcomes is crucial, such as in personalized medicine.

In summary, predictive ability seemed to be enhanced by use of Bayesian neural networks. Due to small sample sizes no claim is made about superiority of any specific non-linear architecture. As it has been observed in many studies, the superiority of one predictive model over another depends on the species, trait and environment, and the same will surely hold for ANNs.

## Abbreviations

ANN: artificial neural network; BR: Bayesian regularization; BRANN: Bayesian regularization artificial neural network; LASSO: Least absolute shrinkage and selection operator; MAP: Maximum a posterior; NN: Neural network; RKHS: Reproducing kernel Hilbert spaces regression; SNP: Single nucleotide polymorphism; SSE: Sum of squared error.

## Competing interests

The authors declare that they have no competing interests.

## Authors' contributions

DG conceived, drafted and wrote the manuscript; HO conceived, carried out the study, performed computations and wrote a part of the manuscript; KAW and GJMR helped to conceive and coordinate the study, provided critical insights and revised the manuscript. All authors read and approved the final manuscript.
